# STAR Suite: Integrating transcriptomics through AI software engineering in the NIH MorPhiC consortium

**DOI:** 10.64898/2026.03.09.710580

**Published:** 2026-03-10

**Authors:** Ling-Hong Hung, Ka Yee Yeung

**Affiliations:** 1 School of Engineering and Technology, University of Washington Tacoma, Tacoma, WA, USA

## Abstract

To accommodate rapid methodological turnover, bioinformatics pipelines typically consist of discrete binaries linked via scripts. While flexible, this architecture relies on intermediate files, sacrificing performance, and treating complex codebases as static silos. For example, the STAR aligner [1]—the standard engine for transcriptomics—uses an external script for adapter trimming, necessitating the decompression and re-compression of large files. These limitations presented scalability problems for uniform processing of data in the NIH MorPhiC consortium. We present our solution, STAR Suite, a human-engineered and AI-implemented modernization that integrates functionality directly into the C++ source. In just four months, a single developer added over 92,000 lines to the original 28,000-line codebase to produce four unified modules: STAR-core, STAR-Flex, STAR-Perturb, and STAR-SLAM **that can be installed as a pre-compiled binary without introducing any new dependencies**. This work demonstrates a new paradigm for the rapid evolution of high-performance bioinformatics software.

## 1 Introduction

Transcriptomics methods have evolved from bulk RNA-seq to complex single-cell chemistries [2], spatial profiling, metabolic and functional assays such as SLAM-seq [3, 4] and Perturb-seq [5, 6]. The STAR aligner [1] remains the standard processing engine for these varied data types in both academic and commercial pipelines, most notably the Cell Ranger [7] and Space Ranger software from 10x Genomics. To manage these workflows, pipelines link discrete binaries via custom scripts and package managers. To avoid the problem of variability in local computational environments, the field adopted containerization orchestrated by workflow managers. Nextflow [8] is the most widely used manager and supports standard workflows maintained by the nf-core community [9]. While effective for reproducibility, this architecture prioritizes encapsulation over integration, allowing legacy technical debt to accumulate in binaries and helpers. Instead of consolidating changes into unified modules, this approach fosters increasingly complex pipelines dependent on fragmented external tools where the interactions become locked in and difficult to update.

Consequently, performant modules such as STAR have become static silos, forcing pipelines to rely on external tools to adapt to changing technology. For instance, nf-core implements adapter trimming using the 15-year-old Python utility Cutadapt [10], necessitating the costly decompression and re-compression of massive files because this simple function is not integrated into the aligner. STAR does not even have a true batch mode for handling multiple samples, relying on scripting multiple STAR calls. STAR does provide the workaround of keeping indices in memory but this does not function in many HPC and Dockerized environments, necessitating the constant reloading of large index files. Even where native STAR functionality exists, the community may bypass it in favor of specialized external binaries. For example, STAR’s internal sorting is frequently substituted with Samtools [11] to minimize disk usage, while quantification is offloaded to Salmon [12] for advanced modeling using Variational Bayes. In addition to the overhead of file sharing or data piping, this fragmentation can also result in technical inconsistency; GrandSLAM [13] uses STAR BAM files to identify new RNA synthesis but must rely on an internal implementation to approximate STAR’s counting algorithm as it cannot access those routines directly. Finally, while STAR added STARsolo [14] to mirror 10x Genomics’ Cell Ranger’s single-cell RNA-seq processing, that was for versions 3 to 5, which has drifted significantly from the current version 9. It also does not support emerging assays like 10x Flex and the feature processing needed by Perturb-seq, where the performance overhead of Python and R renders script-based solutions unviable, leaving the community with no robust open-source alternatives.

The barrier to internal modification is primarily the prohibitive technical scale of the infrastructure. A legacy codebase like STAR—spanning over 28,000 lines across 250 files—presents a formidable challenge; navigating the interdependencies required to safely modify such a system is a massive undertaking. Even implementing a simple internal batch scheduler requires a deep understanding of the data structures to avoid memory leaks. These difficulties are evidenced by the fact that 10x Genomics does not alter STAR, preferring to orchestrate a frozen version with proprietary scripts and binaries for their software. These technical barriers are compounded by academic priorities that favor the creation of novel external modules over the maintenance of existing core infrastructure. While it is possible to publish and obtain funding for a new method, it is extremely difficult to justify the “invisible” work of eliminating technical debt in another group’s tool. Consequently, the field has accepted these architectural compromises as a necessary cost of doing science.

The Molecular Phenotypes of Null Alleles in Cells (MorPhiC) consortium [15] is an NIH Common Fund program that aims to systematically knock out every human protein-coding gene and characterize the resulting molecular phenotypes using diverse transcriptomic assays. Multiple data production centers generate scRNA-seq, Perturb-seq, Flex, and SLAM-seq datasets that must be processed uniformly at scale. Our group was tasked with this processing, and while we have our modern in-house orchestration engine [16], the scale of the data processing quickly highlighted the limitations of the workarounds. Mapping feature barcodes, handling multiple 10x barcode schemes, and handling assays such as Flex necessitated complicated development of in-house software to process the data which were handled inefficiently by orchestration of external methods. With the maturation of AI-assisted software development, it became possible to directly integrate our approaches and modernize STAR directly. The result is STAR Suite, a comprehensive update that integrates new functionalities directly into the C++ source. Using a human-architected, AI-implemented workflow, a single researcher added over 92,000 lines of code to the original 28,000-line base in just four months—a scale of development typically requiring a dedicated engineering team. This effort produced four unified modules—STAR-core, STAR-Flex, STAR-Perturb, and STAR-SLAM (Fig. 3)—backed by a newly implemented suite of regression and unit tests. Despite the expansion in codebase size, STAR Suite adheres to a strict zero-dependency policy, allowing us to provide a pre-compiled binary that replaces the existing STAR binary. This provides all the new capabilities while maintaining all the legacy STAR functions. Partial compile options are provided to allow users to exclude modules that they do not use.

## 2 Results

### 2.1 STAR-core: modernization of STAR for bulk and single-cell RNA-seq restores parity with Cell Ranger 9.0.1

Table 1 summarizes the major changes to core STAR functions and the legacy workarounds each replaces. The integrated adapter trimming (--trimCutadapt) implements the Cutadapt v5.1 [10] algorithm natively in C++, eliminating the decompression–recompression cycle that pipeline approaches require. Native sample batching allows a single STAR invocation to process multiple samples while loading the genome index only once. A spill-to-disk BAM sorting backend (--outBAMsortMethod samtools) reduces temporary disk usage compared to STAR’s legacy bin-based sorter by maintaining bounded in-memory buffers and writing spill files only as needed. Variational Bayes transcript quantification (--quantMode TranscriptVB) provides Salmon-equivalent [12] modeling without an external binary, achieving gene-level Spearman/Pearson correlations of ˜0.99+ on validation datasets. Y-chromosome BAM and FASTQ splitting (--emitNoYBAM, --emitYNoYFastq) was developed for sex-specific analysis of MorPhiC KOLF cell lines, producing separate Y and non-Y outputs during alignment without post-processing. Poly-G trimming (--clip3pPolyG) addresses the poly-G artifact on NovaSeq/NextSeq platforms that inflates counts for specific genes (e.g., LINC00486), restoring gene-level Pearson correlation that was otherwise degraded. Both the trimming and SLAM-seq modules generate self-contained interactive HTML and JSON QC reports (--trimQcReport, --slamQcReport) covering adapter statistics, T>C conversion rate profiles, and auto-trim visualizations, eliminating the need for external QC tools such as FastQC or MultiQC for these steps.

Table 2 summarizes the scRNA-seq updates that restore Cell Ranger 9.0.1 parity. Significant drift between STARsolo and Cell Ranger 9.0.1 had resulted in gene expression Pearson correlations of 0.92 and cell-calling Jaccard of 0.85. STAR-core addresses this through several coordinated changes: EmptyDrops [17] cell calling now uses a CR9-style bootstrapped recovered cells estimation (100 samples) instead of a hardcoded nExpectedCells=3000, improving cell-calling sensitivity. The CB/UB tag injection pathway was unified so that both sorted and unsorted BAM outputs use the same addBAMtags() function, routed through a buffered “no-sort” mode for unsorted output. CB and UB are handled independently—a valid cell barcode is emitted even when the UMI is invalid (status==2). On a full-depth MorPhiC consortium scRNA-seq dataset, these changes restore gene expression Pearson to 0.998 and cell-calling Jaccard to 0.99.

### 2.2 STAR-Perturb: rapid feature assignment for 3–5× speedup of Perturb-seq and cell lineage tracking

Many single-cell assays rely on assigning one or more sets of feature barcodes—knockout perturbations in CRISPR-based Perturb-seq [5], lineage barcodes for clonal tracking [18], or antibody-derived tags for CITE-seq. No efficient open-source tool exists for this application. We developed a feature barcode search engine written in C that uses a bit-counting algorithm for fast Hamming distance computation, comparing sequences 32 bases at a time using hardware popcount instructions. A two-tier hash scheme provides near-instant detection of exact matches and single-mismatch nearmatches before falling back to the full Hamming search. Feature offset auto-detection extracts the barcode position from the pattern column of the feature reference, eliminating a common source of user error.

**Figure 1:**
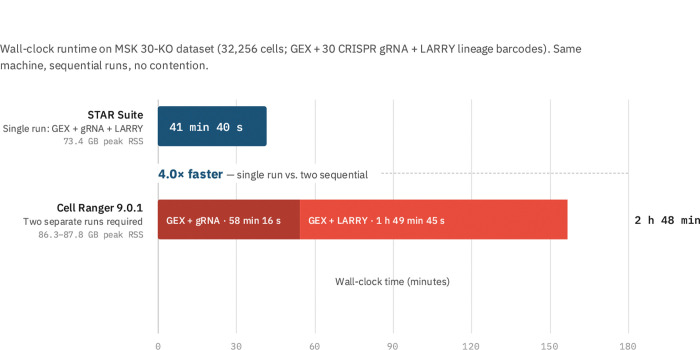
Figure 1. STAR Suite processes three Perturb-seq libraries in a single pass. Wall-clock runtime comparison on the MSK 30-KO Perturb-seq dataset (32,256 cells; GEX, 30 CRISPR gRNA guides, and LARRY lineage barcodes). STAR Suite (dark blue) processes all three libraries in a single 41 min 40 s run. Cell Ranger 9.0.1 cannot process gRNA and lineage barcodes simultaneously, requiring two separate runs: GEX + gRNA (dark red, 58 min 16 s) and GEX + LARRY (light red, 1 h 49 min 45 s), totaling 2 h 48 min — a 4.0× speedup. Peak memory is comparable (STAR Suite 73.4 GB vs. Cell Ranger 86.3–87.8 GB). Despite the architectural difference, quantitative parity is maintained: per-guide Pearson 0.9999, per-cell total UMI Pearson 0.9997, exact call-set match 0.9944. All runs performed sequentially on the same machine (i9–13900KF, 126 GB RAM, 32 threads) with no contention. STAR Suite timing measured via /usr/bin/time −v; Cell Ranger timing from Martian perf high-mem RSS.

In STAR-Perturb, feature barcode searches execute in parallel with genomic alignment. STAR-Perturb also auto-detects the barcode chemistry (NXT vs TRU), and unlike Cell Ranger, supports processing multiple feature libraries (e.g., gRNA and lineage barcodes) in a single run. On the MSK 30-KO Perturb-seq dataset (32,256 cells, 30 CRISPR guides, plus LARRY lineage barcodes),STAR-Perturb processed all three libraries (GEX + gRNA + LARRY) in 41 min 40 s wall time on 32 threads. Cell Ranger required separate runs—58 min 16 s for GEX + gRNA and 1 hr 49 min 45 s for GEX + LARRY—totaling 2 hr 48 min for equivalent output, a 4× speedup (Fig. 1, Table 3).

Quantitative parity is strong. Per-guide Pearson correlation on the merged filtered matrix is 0.9999, with per-cell total guide UMI Pearson of 0.9997 (Table 3). STAR-Perturb recovers 1.044× Cell Ranger’s total guide UMIs, consistent across all 30 guides (range 1.028–1.080×). At the cell-calling level, exact called guide-set match is 0.9944, with mean per-cell Jaccard of 0.9973. Exact singleton match is 0.99995; the residual 0.5% divergence is concentrated in multi-guide cells and reflects STAR-Perturb’s higher sensitivity—STAR recovers guide assignments that Cell Ranger misses even when restricting to exact sequence matches, yielding directional containment of CR-in-STAR = 0.9963 vs. STAR-in-CR = 0.9918.

GMM-based CRISPR feature calling (--crMinUmi) was validated on the A375 dataset (1,083 Cell Ranger cells), achieving 100% exact match on all 1,083 common cells with UMI thresholds within 4% of Cell Ranger values.

### 2.3 STAR-Flex: first open-source 10x Flex scRNA-seq with CMO demultiplexing

STAR-Flex provides the first open-source implementation of the 10x Genomics Fixed RNA Profiling (Flex) workflow. The pipeline builds a hybrid reference genome with synthetic probe pseudo-chromosomes, then uses STAR’s alignment engine to quantify probe hits while using genomic alignments to confirm matches and detect off-probe noise. Sample multiplexing is handled via inline RTL tag detection, and cell calling uses a shared libscrna library implementing EmptyDrops, OrdMag, and occupancy filtering.

The Flex pipeline includes: (i) sample tag detection during alignment; (ii) inline hash capture of CB/UMI/gene tuples; (iii) 1MM pseudocount-based cell barcode correction (Cell Ranger-compatible); (iv) clique-based UMI deduplication; (v) OrdMag or full EmptyDrops cell filtering per sample; and (vi) Monte Carlo occupancy filtering. A standalone run_flexfilter_mex tool allows offline re-running of cell calling with different parameters on existing MEX outputs.

Benchmarking against Cell Ranger v7.1 demonstrates effectively identical quantification (Pearson > 0.999) with cell-calling Jaccard > 0.99. The marginal divergence (<1%) is attributable to stochasticity in the EmptyDrops algorithm and the correction of specific errors identified in the proprietary pipeline, such as the erroneous retention of off-probe genomic reads in the Cell Ranger assignments.

### 2.4 STAR-SLAM: harmonized metabolic labeling

Current best practices for SLAM-seq [3, 19] rely on wrapping STAR with external tools like GEDI/GrandSLAM [13] to identify T>C conversions. This separation creates “logic drift,” as external tools must approximate the aligner’s internal counting decisions. STAR-SLAM eliminates this by performing mutation detection and background modeling directly within the aligner’s critical path.

STAR-SLAM implements full gene-level new-to-total RNA ratio (NTR) estimation using binomial and EM models, producing output in the GrandSLAM column schema for seamless compatibility with downstream tools. SNP handling supports both user-provided BED/VCF masks and an internal auto-detection mode that uses a Kneedle-style knee detection algorithm on the mismatch fraction distribution, with guardrails clamping the threshold to [0.10, 0.60] and a fallback to 0.22 (GEDI-parity value) when auto-estimation fails.

A key innovation is variance-based auto-trimming (--autoTrim variance): rather than relying on Phred quality scores, STAR-SLAM computes the standard deviation of T>C conversion rates at each read position and fits a piecewise linear regression (2–4 segments, selected by BIC) to locate the “knees” where artifact-prone ends transition to clean signal. This directly addresses chemical modification artifacts at read ends that do not correlate with base quality scores.

A standalone slam requant tool, built from the same SLAM quantification engine, enables offline requantification with different auto-trim or SNP parameters on existing BAM files, allowing iterative optimization without re-alignment.

On the GrandSLAM-provided human benchmark (100K reads), STAR-SLAM achieves NTR Pearson of 0.999 with an external SNP mask (384 genes, ≥20 read-count threshold) and 0.990 with internal SNP detection (Table 4). Raw conversion-count (k/nT) Pearson reaches 0.9999 under the BED mask. A GEDI-compatibility mode (--slamCompatMode gedi) mirrors specific GEDI algorithmic choices—intronic classification, lenient overlap acceptance, and overlap-gene weighting—enabling controlled parity testing during migration from existing GEDI workflows. This is a clean-room re-implementation; no GEDI source code was used.

### 2.5 AI-assisted engineering and future-proofing

The rapid modernization of the STAR codebase—expanding from 28,228 lines across 250 files to 120,486 lines across 522 files in under four months—was enabled by a “Human-Architect, AI-Implementer” workflow (Fig. 4a). For each feature, the human researcher collaborated with AI agents to produce an architectural plan specifying module boundaries, data flow, test criteria, and end-to-end gold standards. AI agents then entered an autonomous debug/refine loop: implementing the specified logic, executing both AI-authored unit tests and end-to-end regression tests against the human-defined gold standards, and reviewing their own output for correctness and plan conformance. This loop iterated under human supervision until parity metrics passed. Over 70 architectural plans were produced during development, each serving as a persistent context document that future agents can reference. The development spanned over 300 commits across multiple repositories during this period, with 73 test scripts covering regression, smoke, and unit testing across all four modules.

By decomposing the repository into atomic file-level contexts, a single researcher directed AI agents to implement complex logic—including the Core, Flex, Perturb, and SLAM modules—with high velocity (Fig. 4c). The quality of AI-driven code review improved substantially over the development period, enabling increasingly autonomous operation. The zero-dependency constraint was enforced architecturally: all new functionality (EmptyDrops, OrdMag, BAM sorting, feature barcode search, GMM calling, NTR estimation) was implemented in C/C++ with no external library requirements beyond what upstream STAR already uses.

To prevent this expanded codebase from becoming a new static silo, we integrated a Model Context Protocol (MCP) server directly into the repository (Fig. 4b). This server exposes tools for dataset discovery (list_datasets), pre-flight validation (preflight), script execution (run_script), and output collection (collect_outputs), with path validation against trusted roots and a job queue with timeout handling. An AGENTS.md context file at the repository root provides a structured map of the codebase, build instructions, key technical decisions, and recent changes, enabling AI agents to autonomously navigate the source code and execute the regression test suite. This infrastructure lowers the barrier to community maintenance, allowing researchers to utilize agentic workflows to validate and merge new features without mastering the entire C++ architecture.

## 3 Discussion

STAR Suite demonstrates that direct integration of new functionality into a mature, high-performance C++ codebase is both feasible and advantageous when enabled by AI-assisted software engineering. By consolidating adapter trimming, BAM sorting, Variational Bayes quantification, Flex probe alignment, feature barcode assignment, CRISPR calling, SLAM-seq quantification, and cell calling into a single binary, STAR Suite eliminates the intermediate-file overhead and technical inconsistencies inherent in pipeline-based approaches.

**Figure 2:**
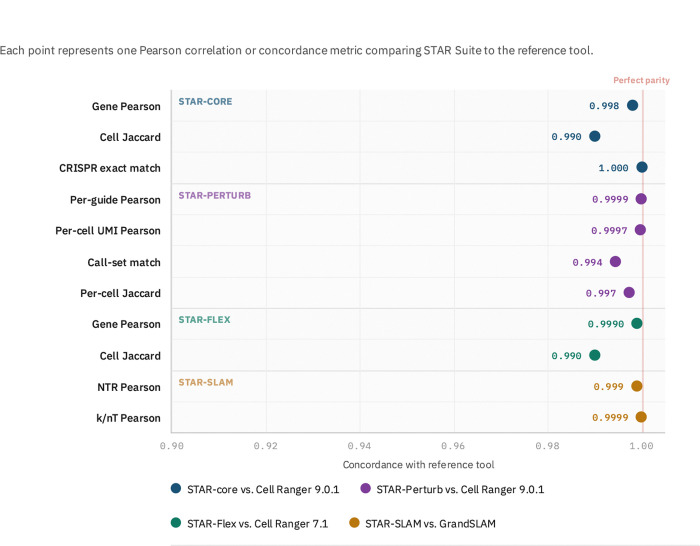
Figure 2. STAR Suite achieves near-perfect quantitative parity across four assay types. Each point represents one concordance metric comparing STAR Suite output to the reference tool. Points are colored by module; the vertical red line marks perfect parity (1.0). All 11 metrics exceed 0.99. **STAR-core** (blue): gene expression Pearson correlation and cell-calling Jaccard index on a full-depth MorPhiC consortium scRNA-seq dataset compared to Cell Ranger 9.0.1; CRISPR exact match rate on 1,083 cells from the A375 10x CRISPR 5’ GEX dataset. **STAR-Perturb** (purple): per-guide Pearson (30 CRISPR guides), per-cell total UMI Pearson, exact called guide-set match, and mean per-cell Jaccard on the MSK 30-KO Perturb-seq dataset (32,256 cells) compared to Cell Ranger 9.0.1. **STAR-Flex** (green): gene expression Pearson and cell-calling Jaccard on JAX Flex datasets compared to Cell Ranger 7.1. **STAR-SLAM** (gold): new-to-total RNA ratio (NTR) Pearson and raw conversion fraction (k/nT) Pearson on the GrandSLAM 100K-read human benchmark with external BED mask at ≥20 reads threshold. See Tables 2–4 for complete numerical values.

**Figure 3:**
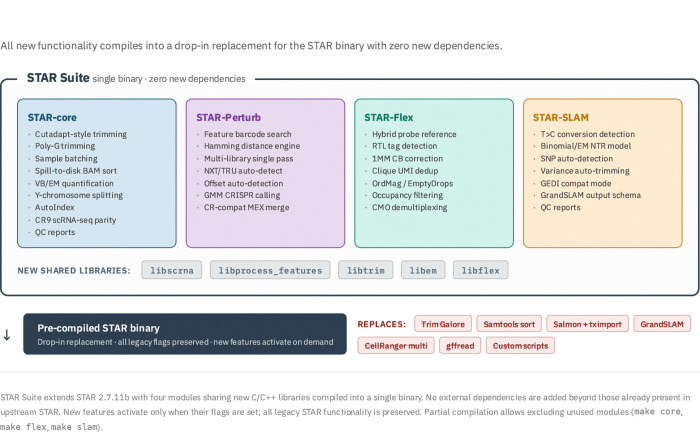
Figure 3. STAR Suite integrates four modules and new shared libraries into a single binary. STAR Suite extends STAR 2.7.11b with four modules — STAR-core (bulk and scRNA-seq modernization), STAR-Perturb (feature barcode assignment and CRISPR calling), STAR-Flex (10x Fixed RNA Profiling), and STAR-SLAM (metabolic labeling) — sharing five new C/C++ libraries: libscrna (EmptyDrops, OrdMag, and occupancy cell calling), libprocess features (bit-parallel Hamming barcode search), libtrim (Cutadapt-style adapter trimming), libem (Variational Bayes/EM transcript quantification), and libflex (Flex pipeline logic). All modules compile into a single pre-compiled binary that serves as a drop-in replacement for the upstream STAR binary with zero new dependencies. The binary replaces Trim Galore (Cutadapt + FastQC), Samtools sort, Salmon + tximport, GrandSLAM, CellRanger multi (the only existing tool for Flex and feature barcode processing), and gffread. New features activate only when their flags are set; all legacy STAR functionality is preserved. Partial compilation options (make core, make flex, make slam) allow users to exclude modules they do not use.

**Figure 4:**
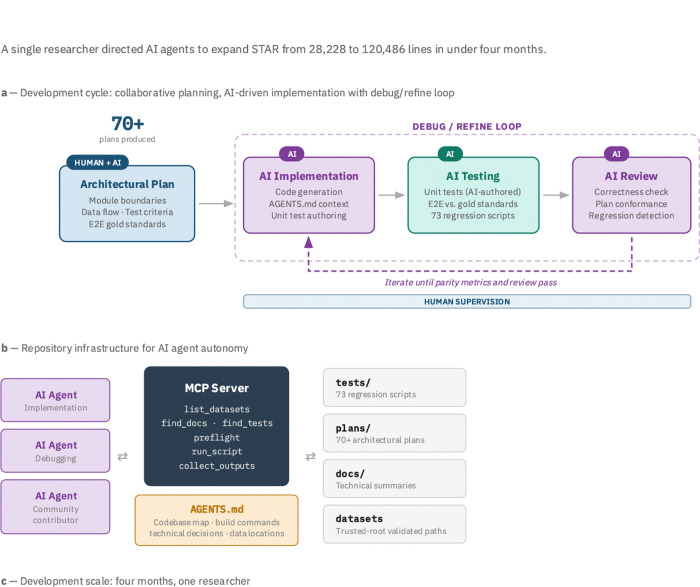
Figure 4. Human-Architect, AI-Implementer workflow for rapid codebase modernization. **a**, Development cycle for each feature. The human researcher collaborates with AI to produce an architectural plan specifying module boundaries, data flow, test criteria, and end-to-end gold standards. AI agents then enter a debug/refine loop (dashed bracket): AI implements the code using the AGENTS.md context file for codebase orientation, AI executes the test suite (both AI-authored unit tests and end-to-end regression tests against human-defined gold standards), and AI reviews the result for correctness, plan conformance, and regressions. This loop iterates autonomously until parity metrics pass, under human supervision. Over 70 architectural plans were produced during development, stored in plans/ as persistent context for future agents. **b,** Repository infrastructure for AI agent autonomy. A Model Context Protocol (MCP) server exposes structured tools for dataset discovery, documentation search, pre-flight validation, script execution, and output collection, with path validation against trusted roots and a job queue with timeout handling. The AGENTS.md context file provides a structured map of the repository layout, build commands, key technical decisions, and data locations, enabling AI agents to navigate the codebase without human guidance. **c,** Development scale over a four-month period (November 2025 – March 2026) with a single researcher. The codebase expanded from 28,228 lines across 250 files (upstream STAR 2.7.11b) to 120,486 lines across 522 files, accumulating over 300 commits across multiple repositories and four modules.

The quantitative benchmarks validate this approach across diverse assay types (Fig. 2). For standard scRNA-seq, gene expression Pearson correlation with Cell Ranger 9.0.1 is restored to 0.998 with cell-calling Jaccard of 0.99 on MorPhiC consortium benchmarks, addressing years of accumulated drift. For Perturb-seq, STAR-Perturb achieves 0.9999 per-guide Pearson with 4× speedup over Cell Ranger by processing multiple feature libraries in a single pass. STAR-Flex provides the first open-source Flex implementation with >0.999 quantification parity. STAR-SLAM achieves NTR Pearson of 0.999 with GrandSLAM while eliminating the logic drift inherent in external wrappers.

The zero-dependency policy is a deliberate architectural choice. Because STAR Suite ships as a single pre-compiled binary that replaces the existing STAR binary, adoption requires no changes to existing infrastructure—no new containers, no additional package installations, and no modifications to workflow managers. All legacy STAR functionality is preserved; new features activate only when their flags are set.

STAR-Flex benchmarks were validated against Cell Ranger v7.1, as this was the version used by The Jackson Laboratory for internal analysis of the Flex datasets at the time of development. The SLAM-seq benchmarks use the publicly available GrandSLAM 100K-read fixture dataset; full-scale validation on diverse SLAM-seq protocols is ongoing as consortium datasets become available.

The MCP server and AGENTS.md infrastructure represent an investment in the long-term maintainability of the codebase. As AI coding agents continue to improve, repositories that provide structured context and validated test suites will be more amenable to community contributions. STAR Suite is designed to be extended: the modular directory structure (core/, flex/, slam/, core/features/) isolates concerns, the shared libscrna library provides reusable cell-calling primitives, and the regression test suite guards against regressions during modification.

STAR Suite is currently deployed as the standard uniform processing pipeline for the NIH MorPhiC consortium [15], processing datasets from data production centers including Memorial Sloan Kettering Cancer Center and The Jackson Laboratory. This is not a one-time modernization but an ongoing program of consolidation. Multiome support is a near-term priority, and we are applying the same approach to Chromap to create Chromap Suite for ATAC-seq and broader multiomic workflows beyond the STAR aligner. Spatial transcriptomics is also under active development through our Fuji [20] and QuPath [21] integrations for whole-slide image analysis, supported by a user-friendly scheduler for hybrid local and cloud execution [16].

More broadly, STAR Suite illustrates a paradigm shift in how high-performance bioinformatics software can be developed and maintained. The barriers that kept legacy codebases static for over a decade—prohibitive scale, deep interdependencies, lack of institutional incentive—are dissolving as AI models mature. The combination of improved code generation and, critically, improved AI-driven code review now allows a single domain expert to direct the equivalent of a full engineering team. This reopens the possibility of direct modification of core open-source tools, reversing the field’s drift toward ever-more-fragmented pipeline architectures. By integrating functionality that previously required six or more external tools into a single binary accessible via familiar command-line flags, STAR Suite lowers the barrier for biologists and core facilities to process diverse transcriptomic assays without specialized pipeline engineering.

This consolidation also has important implications for the emerging paradigm of agentic execution of bioinformatics workflows. As AI agents increasingly orchestrate computational analyses autonomously, the complexity of the tool chain becomes a limiting factor: each external binary, intermediate file format, and version dependency is an additional failure mode that an agent must navigate. A single binary with a unified flag interface radically simplifies the tool calls an agent needs to make, reducing a multi-step pipeline to a single invocation. The MCP server and AGENTS.md infrastructure described here were designed with this future in mind, providing the structured context that AI agents require to discover, validate, and execute analyses without human intervention. The architectural investments—shared libraries, structured agent context, comprehensive regression suites—ensure that this expanded codebase remains modifiable as both the science and the AI tooling continue to advance. The software, documentation, and architectural plans are available at https://github.com/morphic-bio/STAR-suite.

## Online Methods

### Software architecture

STAR Suite extends STAR 2.7.11b by organizing the codebase into module-focused directories while maintaining a single source of truth for the STAR core in core/legacy/. New functionality is organized into core/features/ (shared overlays: VB/EM quantification, Y-chromosome removal, BAM sorting, feature barcode tools, and libscrna), flex/ (Flex pipeline), and slam/ (SLAM-seq). A top-level Makefile exposes per-module build targets (make core, make flex, make slam). When all modules are compiled, the result is a single STAR binary; when new flags are not invoked, behavior is identical to upstream STAR 2.7.11b.

The zero-dependency constraint means all new functionality—EmptyDrops cell calling, OrdMag filtering, occupancy estimation, BAM sorting, feature barcode searching, GMM-based CRISPR calling, Variational Bayes quantification, and NTR estimation—is implemented in C/C++ using only the standard library and the third-party headers already present in upstream STAR (htslib for BAM I/O, opal for SIMD alignment). Three small header-only libraries were added: PCG random number generation (3,623 lines), khash (617 lines), and kseq (FASTQ parsing).

### Adapter trimming

STAR Suite implements the Cutadapt v5.1 [10] trimming algorithm natively in C++ (--trimCutadapt Yes), providing perfect parity with Trim Galore [22] for quality and adapter trimming without requiring decompression and recompression of FASTQ files. For legacy datasets processed with Trim Galore + cutadapt 3.2, a compatibility mode (--trimCutadaptCompat Cutadapt3) reproduces the older algorithm’s behavior. The trimming module (libtrim/, 962 lines) is a general-purpose feature usable with any STAR workflow.

### BAM sorting

A spill-to-disk coordinate sorting backend (--outBAMsortMethod samtools, 584 lines) uses bounded in-memory buffers (configurable via --limitBAMsortRAM) and writes spill files only when the RAM cap is exceeded. Final output is produced by k-way merge of spill files, eliminating the large temporary files created by STAR’s legacy bin-based sorter.

### Variational Bayes transcript quantification

TranscriptVB (--quantMode TranscriptVB) implements Variational Bayes (default) and EM algorithms for transcript-level abundance estimation within the aligner (libem/, 4,873 lines across 26 files). Library type auto-detection, fragment length distribution modeling, and tximport-style [23] gene-level summarization (--quantVBgenesMode Tximport) are included. Transcriptome FASTA generation during index building (--genomeGenerateTranscriptome Yes) eliminates the need for separate gffread or rsem-prepare-reference runs.

### Cell calling (libscrna)

A shared C library (libscrna/) provides EmptyDrops [17], OrdMag, and occupancy-based cell filtering used by both the non-Flex scRNA-seq and Flex pipelines. The non-Flex path uses CR9-style bootstrapped recovered cells estimation (100 samples) for adaptive cell recovery, with 100K Monte Carlo simulations for EmptyDrops and BH FDR correction. The Flex path retains fixed nExpectedCells=3000 (Cell Ranger 7.1 defaults) with 10K simulations and raw p-value thresholds.

### Unsorted BAM CB/UB injection

Both sorted and unsorted BAM outputs now use the same tag injection function (addBAMtags()). For unsorted output, records are buffered via a global SamtoolsSorter instance operating in “no-sort” mode, maintaining FIFO order during mapping. After Solo counting completes, records are streamed back with tags injected. This eliminates the previous two-pass injection path and ensures consistent tag content regardless of output mode.

### Feature barcode assignment

The feature barcode engine (process_features, vendored in core/features/) implements a two-tier search: (i) hash-based lookup for exact and 1-mismatch matches, providing near-instant detection; (ii) bit-parallel Hamming distance computation using hardware popcount instructions, comparing 32 bases per operation. Feature offset is auto-detected from the pattern column of the feature reference CSV; if multiple offsets are detected (>5% heterogeneity), the tool errors with guidance to use --feature_constant_offset or --force-individual-offsets.

### GMM-based CRISPR feature calling

When processing --crMultiConfig with CRISPR Guide Capture features, STAR automatically runs Gaussian Mixture Model (GMM)-based feature calling after EmptyDrops filtering. The GMM determines per-guide UMI thresholds for assigning guide calls to cells. The minimum UMI threshold (--crMinUmi, default 10) is configurable for different assay types: 10 for CRISPR guides (variable capture efficiency), 2–3 for lineage barcodes (stable features). Output files (crispr _analysis/) match the Cell Ranger format.

### STAR-Flex implementation

STAR-Flex implements the 10x Genomics Fixed RNA Profiling workflow via a hybrid reference genome containing synthetic chromosomes for each probe sequence. During alignment, probe hits are quantified using STAR’s alignment engine while genomic hits confirm matches and detect off-probe noise. The downstream pipeline diverges from standard STARsolo due to the presence of RTL sample tags on the same mate as the probe (not the cell barcode mate), which precludes use of STAR’s native barcode/UMI correction routines.

The Flex-specific path (9,670 lines across 25 files) includes: inline hash capture of CB/UMI/gene tuples; 1MM pseudocount-based CB correction (Cell Ranger-compatible); clique-based 1MM UMI deduplication; per-sample OrdMag or full EmptyDrops cell filtering; and Monte Carlo occupancy filtering. Integrated probe reference building filters probes by GTF gene match, validates 50bp A/C/G/T-only sequences, and produces the hybrid genome index in a single --runMode genomeGenerate invocation.

### STAR-SLAM implementation

STAR-SLAM performs T>C conversion detection, SNP masking, auto-trimming, and gene-level NTR estimation within the aligner’s read-processing loop. For each aligned read, genomic positions are inspected for T>C conversions (sense strand), with positions matching known SNPs excluded. The NTR is estimated per gene using a binomial model with EM inference, computing Maximum A Posteriori (MAP) and mean estimates with 0.05/0.95 quantile credible intervals from Beta posterior parameters.

SNP handling offers two modes: (i) external BED/VCF mask (--slamSnpBed) for samples with known variants; (ii) internal auto-detection (--slamSnpDetect 1) using a Kneedle-style knee detection algorithm on the per-position mismatch fraction distribution. The auto-detection builds a histogram of mismatches/coverage at positions with ≥10 coverage, applies log1p normalization, and finds the bin maximizing distance from the diagonal. Guardrails require ≥1,000 eligible sites, knee strength >0.02, and clamp the result to [0.10, 0.60], with fallback to 0.22 (GEDI-parity value).

Variance-based auto-trimming (--autoTrim variance) analyzes the standard deviation of T>C conversion rates across read positions. A piecewise linear regression (2, 3, or 4 segments, selected by BIC) is fitted to the smoothed standard deviation curve; breakpoints define the reliable region boundaries. This addresses chemical modification artifacts at read ends that do not correlate with Phred quality scores. Trim values can be computed once from the first input file and applied globally (--trimScope first, default) or independently per file (--trimScope per-file).

A GEDI-compatibility mode (--slamCompatMode gedi) enables four specific behaviors for parity testing: (i) intronic classification of unspliced reads overlapping introns of multiple transcripts; (ii) lenient overlap acceptance (≥50% exon overlap + splice junction concordance); (iii) read-level overlap-gene weighting (weight = 1/nTr/geneCount); (iv) configurable position filtering for PE overlap and trim guards. All compat features use sentinel-value override semantics, allowing individual behaviors to be toggled independently.

### Standalone utilities

In addition to the main STAR binary, STAR Suite provides standalone tools that link the same shared libraries used by the integrated pipeline, exposing module functionality for offline use without re-alignment. slam_requant compiles the STAR Suite SLAM quantification engine (SlamQuant, SlamSolver, SlamCompat, SlamVarianceAnalysis) into a standalone binary that performs offline SLAM-seq requantification on existing BAM files, allowing users to iterate on SNP masks, auto-trim settings, and model parameters without re-aligning. run_flexfilter_mex links libflex and libscrna to re-run Flex cell calling (OrdMag, EmptyDrops, occupancy filtering) with different parameters on existing MEX outputs. assignBarcodes links libprocess_features and libscrna to run feature barcode assignment, EmptyDrops cell calling, and MEX output as a standalone tool outside of the STAR alignment pipeline.

### QC reporting

Both the trimming and SLAM-seq modules produce self-contained interactive QC reports in HTML and JSON format. Trim QC reports (--trimQcReport) include adapter match rates, trimmed length distributions, and quality score profiles. SLAM QC reports (--slamQcReport) visualize per-position T>C conversion rates, the auto-trim variance curve with fitted breakpoints, SNP detection histograms, and per-gene NTR distributions. These reports are generated without external dependencies (no FastQC, MultiQC, or R installation required) and can be viewed directly in a web browser.

### Benchmarking datasets

STAR-core scRNA-seq parity was validated on full-depth scRNA-seq datasets generated by MorPhiC consortium data production centers, as well as the publicly available A375 10x CRISPR 5’ GEX dataset. STAR-Perturb was benchmarked on the MSK 30-KO Perturb-seq dataset (GEX + 30 CRISPR guides + LARRY lineage barcodes, produced by the Huangfu Lab at Memorial Sloan Kettering Cancer Center) and the A375 CRISPR dataset (11 features, 1,083 Cell Ranger-called cells). STAR-Flex was validated against Cell Ranger v7.1 on JAX Flex datasets. STAR-SLAM was benchmarked against GrandSLAM using the publicly available GrandSLAM 100K-read human fixture dataset with reference NTR values.

### Benchmarking metrics

Gene expression parity was assessed by Pearson and Spearman correlation on log-transformed counts for genes exceeding a minimum count threshold. Cell-calling overlap was assessed by Jaccard index on the called cell sets. Feature barcode parity was assessed by per-guide Pearson correlation on UMI counts, per-cell total UMI Pearson, exact called guide-set match rate, and directional containment metrics (CR-in-STAR and STAR-in-CR). SLAM-seq parity was assessed by Pearson and Spearman correlation on NTR and raw conversion fractions (k/nT) at multiple read-count thresholds (≥20, ≥50, ≥100). Runtime comparisons used wall-clock time from /usr/bin/time −v for STAR and Martian _perf for Cell Ranger, on the same machine (i9–13900KF, 126 GB RAM, 32 threads) with sequential runs to avoid contention.

### AI-assisted development workflow

Development followed a “Human-Architect, AI-Implementer” paradigm. For each feature, the human researcher collaborated with AI agents (Claude, via Cursor IDE) to produce an architectural plan specifying: module boundaries and file ownership; data structures and memory management; integration points with existing code; expected test criteria and parity metrics; and end-to-end gold standards for validation. AI agents then entered a debug/refine loop—implementing the specified logic, authoring and executing unit tests, running end-to-end regression tests against human-defined gold standards, and reviewing their own output for correctness and plan conformance—iterating under human supervision until parity metrics passed. Over 70 plans were produced during the four-month development period, stored in plans/ as persistent context for future agents.

### MCP server and AGENTS.md

A Model Context Protocol (MCP) server provides structured tooling for AI agent workflows: dataset discovery (list_datasets), documentation search (find_docs, find_tests), pre-flight validation (preflight), script execution (run_script with allowlisting), and output collection (collect_outputs). All paths are validated against trusted roots. A job queue (1 concurrent, 10 queued) with timeout handling and process group cleanup prevents runaway executions.

The AGENTS.md context file at the repository root provides a structured map of the repository layout, build commands, key technical decisions (with links to detailed summaries), data locations, branching policy, and CI/CD configuration. This enables AI agents to orient in the codebase without human guidance, reducing the barrier to community contributions.

### Testing infrastructure

The test suite comprises 73 scripts organized by module: CB/UB regression tests, CR-compat parity tests, CRISPR feature calling integration tests, Flex smoke and multi-sample tests, SLAM fixture parity and E2E tests, Y-chromosome splitting tests, parameter regression tests, and TranscriptVB/Salmon parity tests. CI/CD uses GitHub Actions with path-filtered workflows: pull requests run fast checks (build + Tier A smoke); pushes to dev run integration checks; pushes to master publish multi-architecture Docker images (amd64 + arm64); tags trigger release pipelines.

## Figures and Tables

**Table 1:** STAR-core bulk and general updates and the workarounds they replace.

Feature	STAR Suite Flag	Legacy Workaround	Benefit
Adapter trimming	--trimCutadapt Yes	External Cu- tadapt/Trim Galore (decompress → trim → recompress)	Eliminates I/O round-trip; cutadapt v5.1 parity
Poly-G trimming	--clip3pPolyG auto	No workaround (artifacts inflate gene counts)	Removes NovaSeq/NextSeq poly-G artifacts
Sample batching	Native multi-sample mode	Scripted sequential STAR calls, each reloading genome index	Single index load for all samples
BAM sorting	--outBAMsortMethod samtools	Piping to external Samtools sort	Spill-to-disk; bounded RAM; no intermediate files
Transcript quantification	--quantMode TranscriptVB	External Salmon alignment-mode + tximport	VB/EM within aligner; gene-level Pearson ˜0.99+ vs Salmon
Y-chromosome splitting	--emitNoYBAM yes, --emitYNoYFastq yes	Post-hoc BAM filtering with external tools	Inline splitting during alignment; BAM + FASTQ outputs
Transcriptome FASTA	--genomeGenerateTranscriptome Yes	External gffread/rsemprepare-reference	Generated at index time; Salmon- compatible format
AutoIndex	--autoIndex Yes	Manual download, formatting, and index building	Automated download + checksum + CellRanger-style formatting
QC reports	--trimQcReport, --slamQcReport	External FastQC/MultiQC	Self-contained HTML + JSON; no external dependencies

**Table 2:** STAR-core scRNA-seq updates restoring Cell Ranger 9.0.1 parity.

Feature	STAR Suite Change	CR9 Behavior Matched	Parity Metric
Cell calling (EmptyDrops)	Bootstrapped recovered_cells (100 samples) via libscrna	CR9-style adaptive cell recovery	Jaccard 0.99
CB/UB tag injection	Unified sorted/unsorted path via addBAMtags() + noSort buffer	Consistent tag injection regardless of output mode	Bit-identical tags
CB/UB independence	CB valid → emit CB even if UMI invalid (status==2)	Independent CB and UB handling	CB/UB tag agreement
UMI deduplication	--soloUMIdedup 1MM_CR	CR-style 1MM collapse	
UMI filtering	--soloUMIfiltering MultiGeneUMI CR	CR-style multi-gene UMI filter	
GEX feature source	--soloCrGexFeature gene	Exon-only counting (Gene, not GeneFull)	Gene Pearson 0.998
CR-compat MEX merge	--crMultiConfig	Merged raw + filtered MEX with per-library outputs	Format-identical MEX
CRISPR feature calling	--crMinUmi 10 + GMM	Automatic GMM calling on Guide Capture features	100% exact match

**Table 3:** STAR-Perturb benchmarking against Cell Ranger 9.0.1 on MSK 30-KO Perturb-seq.

Metric	STAR-Perturb	Cell Ranger	
**Runtime** Wall time (GEX + gRNA + LARRY)	41 min 40 s	2 hr 48 min (two runs)	4× speedup
Peak RSS	73.4 GB	86.3–87.8 GB	

**Guide count parity (merged filtered, 30 guides)** Total guide UMIs	3,247,179	3,111,361	1.044×
Guide-positive cells	30,263	32,017	
Per-guide Pearson			0.9999
Per-cell total UMI Pearson			0.9997

**Call concordance (23,340 shared called cells)** Exact called guide-set match			0.9944
Exact singleton match			0.99995
Mean per-cell Jaccard			0.9973
CR-in-STAR containment			0.9963
STAR-in-CR containment			0.9918

**Table 4:** STAR-SLAM parity with GrandSLAM on 100K-read human benchmark.

SNP Mode	Threshold	Genes	NTR Pearson	NTR Spearman	k/nT Pearson
External BED mask	≥20 reads	384	0.9989	0.9810	0.9999
External BED mask	≥50 reads	76	0.9961	0.9867	0.9984
External BED mask	≥100 reads	23	0.9944	0.9941	0.9968
Internal detection	≥20 reads	384	0.9898	0.9678	0.9915
Internal detection	≥50 reads	76	0.9963	0.9803	0.9981
Internal detection	≥100 reads	23	0.9937	0.9911	0.9963

## Data Availability

The GrandSLAM benchmark fixture dataset is publicly available at https://github.com/erhard-lab/gedi/wiki/GRAND-SLAM. The A375 10x CRISPR 5’ GEX dataset is publicly available from 10x Genomics. MorPhiC consortium datasets used for internal validation will be available through the MorPhiC Data Explorer upon consortium data release. Benchmark comparison scripts and documentation are included in the repository under tests/ and docs/.
